# The Use of Pointwise Encoding Time Reduction With Radial Acquisition MRA to Assess Middle Cerebral Artery Stenosis Pre- and Post-stent Angioplasty: Comparison With 3D Time-of-Flight MRA and DSA

**DOI:** 10.3389/fcvm.2021.739332

**Published:** 2021-09-09

**Authors:** Feifei Zhang, Yuncai Ran, Ming Zhu, Xiaowen Lei, Junxia Niu, Xiao Wang, Yong Zhang, Shujian Li, Jinxia Zhu, Xuemei Gao, Mahmud Mossa-Basha, Jingliang Cheng, Chengcheng Zhu

**Affiliations:** ^1^Department of Magnetic Resonance, The First Affiliated Hospital of Zhengzhou University, Zhengzhou, China; ^2^Department of Intervention, The First Affiliated Hospital of Zhengzhou University, Zhengzhou, China; ^3^MR Collaboration, Siemens Healthcare, Ltd., Beijing, China; ^4^Department of Radiology, University of Washington, Seattle, WA, United States

**Keywords:** middle cerebral artery, pointwise encoding time reduction with radial acquisition, stent angioplasty, magnetic resonance imaging, digital subtraction angiography

## Abstract

**Background and Purpose:** 3D pointwise encoding time reduction magnetic resonance angiography (PETRA-MRA) is a promising non-contrast magnetic resonance angiography (MRA) technique for intracranial stenosis assessment but it has not been adequately validated against digital subtraction angiography (DSA) relative to 3D-time-of-flight (3D-TOF) MRA. The aim of this study was to compare PETRA-MRA and 3D-TOF-MRA using DSA as the reference standard for intracranial stenosis assessment before and after angioplasty and stenting in patients with middle cerebral artery (MCA) stenosis.

**Materials and Methods:** Sixty-two patients with MCA stenosis (age 53 ± 12 years, 43 males) underwent MRA and DSA within a week for pre-intervention evaluation and 32 of them had intracranial angioplasty and stenting performed. The MRAs' image quality, flow visualization within the stents, and susceptibility artifact were graded on a 1–4 scale (1 = poor, 4 = excellent) independently by three radiologists. The degree of stenosis was measured by two radiologists independently on DSA and MRAs.

**Results:** There was an excellent inter-observer agreement for stenosis assessment on PETRA-MRA, 3D-TOF-MRA, and DSA (ICCs > 0.90). For pre-intervention evaluation, PETRA-MRA had better image quality than 3D-TOF-MRA (3.87 ± 0.34 vs. 3.38 ± 0.65, *P* < 0.001), and PETRA-MRA had better agreement with DSA for stenosis measurements compared to 3D-TOF-MRA (*r* = 0.96 vs. *r* = 0.85). For post-intervention evaluation, PETRA-MRA had better image quality than 3D-TOF-MRA for in-stent flow visualization and susceptibility artifacts (3.34 ± 0.60 vs. 1.50 ± 0.76, *P* < 0.001; 3.31 ± 0.64 vs. 1.41 ± 0.61, *P* < 0.001, respectively), and better agreement with DSA for stenosis measurements than 3D-TOF-MRA (*r* = 0.90 vs. *r* = 0.26). 3D-TOF-MRA significantly overestimated the stenosis post-stenting compared to DSA (84.9 ± 19.7 vs. 39.3 ± 13.6%, *p* < 0.001) while PETRA-MRA didn't (40.6 ± 13.7 vs. 39.3 ± 13.6%, *p* = 0.18).

**Conclusions:** PETRA-MRA is accurate and reproducible for quantifying MCA stenosis both pre- and post-stenting compared with DSA and performs better than 3D-TOF-MRA.

## Introduction

Ischemic stroke is one of the top sources of morbidity and mortality globally (1). Intracranial arterial stenosis is a major cause of ischemic stroke and the incidence of middle cerebral artery (MCA) stenosis in patients with stroke is 7.0–17.7% (2). The degree of stenosis is a critical factor for the management of stroke patients with MCA stenosis. Patients with high-grade stenosis (>70%) have a high risk of recurrent stroke and aggressive medical treatment or endovascular intervention is normally recommended for these patients (3). Digital subtraction angiography (DSA) is considered the gold standard for measuring stenosis, however, it is an invasive technique with radiation exposure, risk of stroke, and contrast-related complications (4). Other non-invasive techniques, including computed tomography angiography (CTA) and contrast, enhanced magnetic resonance angiography (CE-MRA), provide accurate stenosis measurements, but are limited due to radiation exposure and/or contrast-related complications (5, 6). Time-of-flight (TOF) is a non-contrast MRA technique that is commonly used for cerebral vascular imaging, but it has limited accuracy due to flow-related artifacts (7).

While the SAMMPRIS trial (8) showed intracranial stenting is not superior to medical therapy, the use of stenting for MCA stenosis remains controversial. The WEAVE trail (9) recently demonstrated the advantage of stenting over medical therapy in select patient populations. An accurate evaluation of stenosis is needed for appropriate patient selection for angioplasty and stenting (10, 11). Post-stenting arterial segment evaluation is also important considering the re-stenosis rate after stenting is 11.9–17.9% (12). This evaluation, however, is challenging for CTA, CE-MRA, and 3D-TOF-MRA due to beam hardening artifacts or magnetic susceptibility artifacts (13–15).

Pointwise Encoding Time Reduction with Radial Acquisition (PETRA) is a non-contrast MRA technique that uses Arterial Spin Labeling (ASL) and UTE techniques (16). The ultra-short TE of <100 μs significantly reduces susceptibility artifact (17). It has been successfully used for the evaluation of intracranial aneurysms after stent-assisted coil embolization (18–20) and the evaluation of other cerebral artery diseases (21, 22). Whether PETRA-MRA can be used as an alternative angiographic technique that approximates DSA performance has not been investigated. We hypothesize that PETRA-MRA can evaluate MCA stenosis more accurately than 3D-TOF-MRA when compared to DSA for both pre-intervention evaluation and post-intervention follow-up.

## Materials and Methods

### Study Population

This study was approved by the local institutional review board, and informed consent was obtained from all participants. Written informed consent was obtained from the individuals for the publication of any potentially identifiable images or data included in this article. Patients who presented between October 2018 and August 2019 were recruited. The inclusion criteria were as follows: (1) patients with stroke or TIA attributing to MCA atherosclerotic stenosis; (2) over 18 years old; (3) underwent MRA that included PETRA-MRA and 3D-TOF-MRA; (4) underwent DSA, with the interval between the MRA and DSA being <1 week. Patients were excluded if they had any of the following conditions: (1) intracranial hemorrhage or non-atherosclerotic intracranial vasculopathy; (2) inadequate image quality; (3) no stenosis identified; (4) incomplete imaging dataset.

### Image Acquisition

Patients with MCA stenosis underwent 3D-TOF-MRA, PETRA-MRA, and DSA examinations within a week to identify patients that were suitable for intracranial angioplasty and stenting treatment. For patients who were selected for intervention, DSA was again performed immediately after the stent placement, and patients then underwent 3D-TOF-MRA and PETRA-MRA within a week of intervention.

### MRI Protocol

Both MRAs were performed on a 3-Tesla system (MAGNETOM Prisma, Siemens Healthcare, Erlangen, Germany) using a 64-channel head-neck coil.

The scan parameters for 3D-TOF-MRA were: TR/TE, 20/3.69 ms; flip angle, 18°; field of view (FOV), 200 × 160 mm^2^; matrix, 320 × 256; slice thickness, 0.6 mm; voxel size, 0.6 × 0.6 × 0.6 mm^3^; number of slab, 4; slices per slab, 44; acquisition time, 3 min 29 s. The scan parameters of PETRA-MRA were: TR/TE, 3.32/0.07 ms; flip angle, 3°; FOV, 300 × 300 mm; matrix, 320 × 320; radial spoke, 60,000; slice thickness, 0.9 mm; voxel size, 0.9 × 0.9 × 0.9 mm^3^; number of slab, 1; slices per slab, 320; A control scan (bright blood) without the slice-slective saturation slab was first acquired with an acquisition time of 3 min and 29 s, and then a labeled scan (black blood) with a saturation band proximal to the imaging volume was acquired with an acquisition time of 5 min and 51 s. The final images were subtracted from the two datasets (Control-Label).

### Digital Subtraction Angiography

Patients underwent cerebral angiography examinations on a fixed digital angiographic system, FD 20 Artis (Phillips Healthcare, Best, The Netherlands). All procedures were performed with local anesthesia. Femoral arterial access was used in all cases. DSA acquisition protocol was performed with Omnipaque 300 contrast injection (GE Healthcare, Waukesha, WI, USA), at a rate of 4 ml/s. During the 5-s acquisition after a delay of 1 s, a 200-degree rotation of the C-arm was performed to obtain 133 frames. Scan parameters were: FOV = 320 × 320 mm^2^, matrix = 1,024 × 1,024. Four-vessel angiography was performed in all patients. Standard anteroposterior, oblique, and lateral views were obtained for all interrogated arteries.

### Image Analysis

The maximum intensity projection (MIP) of both MRA sequences were reconstructed by a neuroradiologist using a dedicated workstation. The MRA datasets were anonymized and placed in random order. Both the source images and MIPs were used for image evaluation. Three experienced radiologists (observer A, 8 years of experience; observer B, 5 years of experience; observer C, 5 years of experience) reviewed the image quality of the MRAs independently and blindly (for example, when evaluating PETRA-MRA, 3D-TOF-MRA images were not present), and two experienced radiologists (observer A, 8 years of experience; observer B, 5 years of experience) measured the degree of stenosis on the MIPs of MRAs and DSA independently and blindly (without knowing the patients' clinical information, and when evaluating one imaging modality, without seeing other modality's images).

For pre-intervention MRAs, the overall image quality was reported using a previously defined four-point scale to determine signal homogeneity, lesion conspicuity, quality of venous signal suppression, and diagnostic confidence (21): 4 = excellent (excellent quality diagnostic information with detailed vascular architecture, no artifacts), 3 = good (good quality diagnostic information with adequate delineation of the vascular architecture, minimal artifacts), 2 = poor (poor quality diagnostic information with ordinary delineation of the vascular architecture, moderate artifacts), and 1 = not visible (almost no signal of the vascular architecture, severe artifacts).

After the intervention, two qualitative image scores were used to evaluate the susceptibility artifact and in-stent flow signal which were adopted from a previous publication (19). The ratings of susceptibility artifact intensity were determined using the following four-point scale: 4 = no susceptibility signal loss; 3 = minimal signal loss; 2 = moderate signal loss, which compromised image assessment; and 1 = severe signal loss, which prevented image assessment. The ratings of the in-stent flow signal were determined using the following four-point scale: 4 = excellent (excellent quality diagnostic information, nearly equal to that of DSA); 3 = good (good quality diagnostic information with minimal blurring or artifacts); 2 = poor (structures were slightly visible but with significant blurring or artifacts, not diagnostic); and 1 = arterial segments not visible (almost no signal).

The degree of stenosis was measured according to WASID criteria (23):

Stenosis%=(1−d/D)×100%

the *d* is the diameter of the lumen at the most stenotic site, and the *D* is the diameter of the lumen at the proximal normal segment.

### Statistical Analyses

All statistical analyses were performed using SPSS 17.0 software or GraphPad Prism 5. Continuous variables were expressed as the means ± standard deviation (SD). The image quality scores were compared using the Wilcoxon signed-rank test. Spearman's correlation coefficient (*r*) was used as a non-parametric test to evaluate the linear association between measurements of stenosis on MRA and DSA.

The agreement of stenosis measurements between MRA and DSA and the inter-reader agreement was assessed using Bland–Altman analysis and intra-class correlation coefficient (ICC) (24), and the bias and limit of the agreement were reported. Measurement error was quantified by the coefficient of variation (CV) (CV = SD of difference/mean × 100%).

The performance of PETRA-MRA and 3D-TOF-MRA for the detection of stenosis >50% and stenosis >75% was summarized by the sensitivity, specificity, positive predictive value (PPV), and negative predictive value (NPV), using DSA as the reference standard. *P* < 0.05 indicated a significant difference and all *p*-values were two-sided.

## Results

### Patient Demographics and Imaging Findings

**Figure 1:**
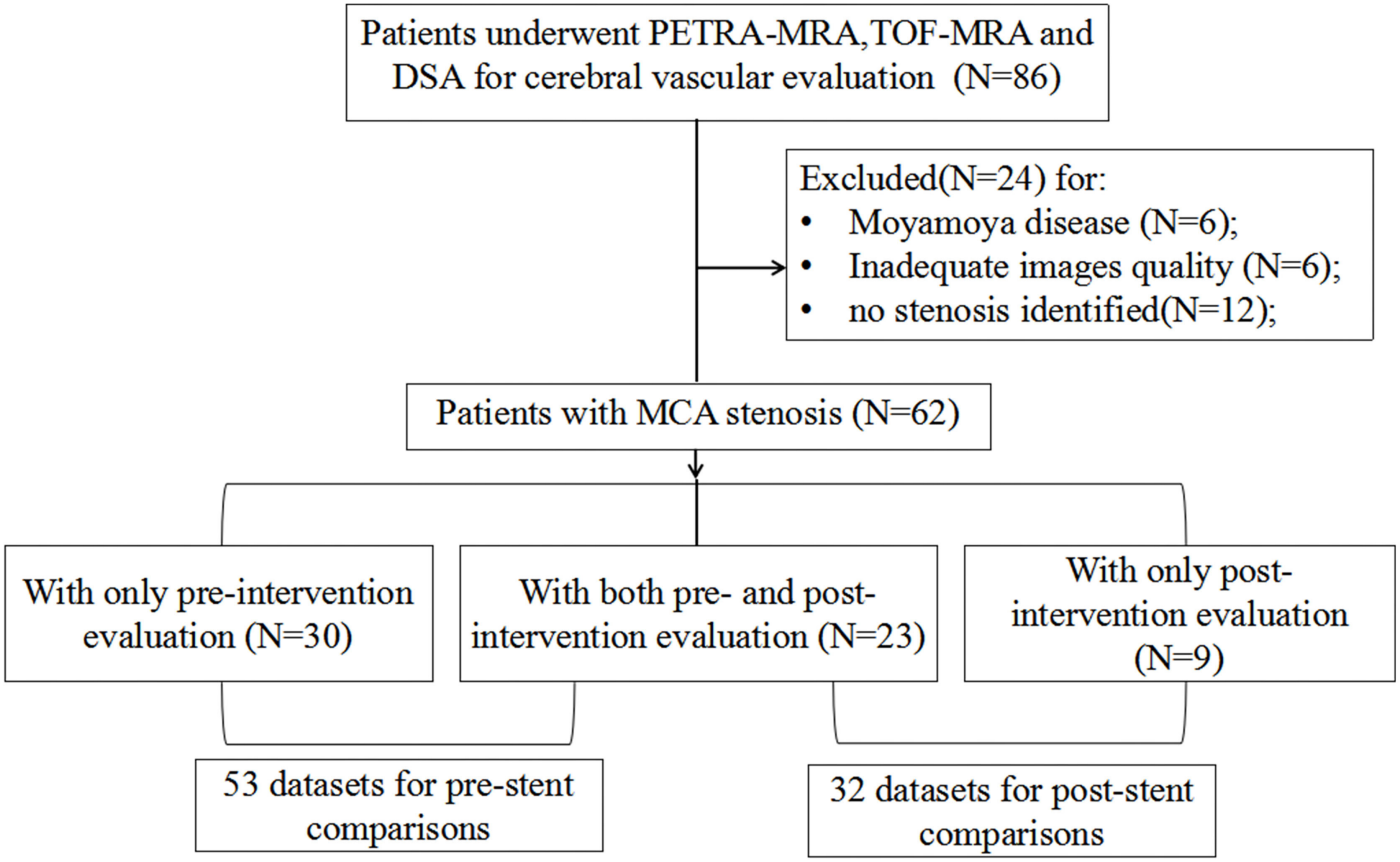
Patient selection flowchart.

From an initial cohort of 86 patients, 24 patients have excluded: 6 patients had Moyamoya disease, 6 patients had image degradation due to motion artifact, and 12 patients had no intracranial stenosis identified. This left 62 patients for inclusion in the final analysis. The patient selection flow chart is shown in Figure 1. Of these patients, 30 patients had an only pre-intervention evaluation, 23 patients had both pre- and post-intervention evaluation, and 9 patients had only post-intervention evaluation, resulting in 53 datasets for pre-intervention comparison and 32 datasets for post-intervention comparison.

The time interval between MRI and DSA was 3.8 ± 2.4 days for pre-intervention comparison, and the interval was 3.7±1.0 days for post-intervention comparison. Thirtty-five patients had a stroke and 27 patients had a transient ischemic attack (TIA). The interval between the initial symptom onset and MRI was 41.7 ± 48.5 days, and the interval between symptom onset and DSA was 44.9 ± 48.1 days.

For pre-intervention evaluation, PETRA-MRA had better image quality than 3D-TOF-MRA (3.87 ± 0.34 vs. 3.38 ± 0.65, *P* < 0.001). For post-intervention evaluation, PETRA-MRA had better in-stent flow visualization (3.34 ± 0.60 vs. 1.50 ± 0.76, *P* < 0.001) and less susceptibility artifact (3.31 ± 0.64 vs. 1.41 ± 0.61, *P* < 0.001).

**Figure 2:**
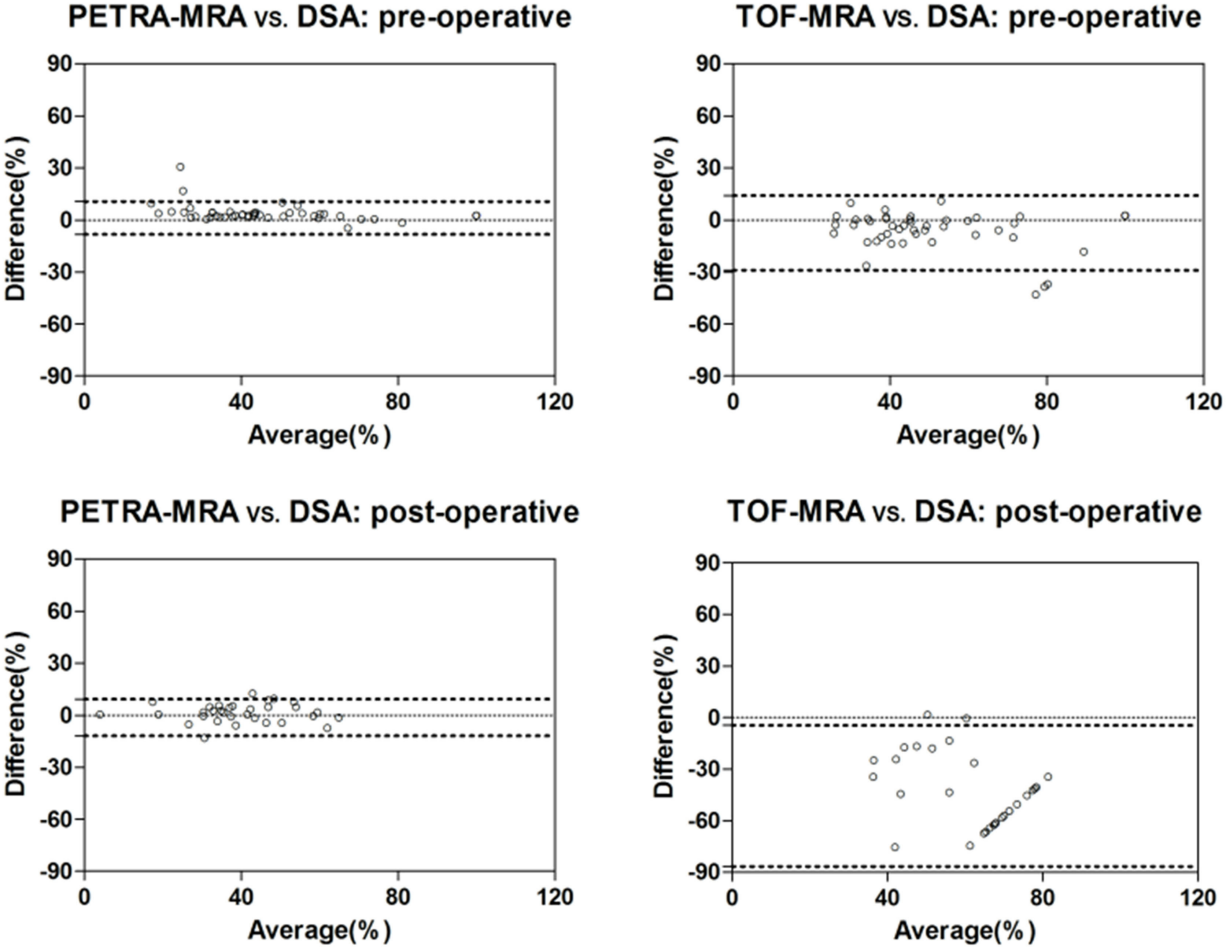
Bland-Altman plots for the comparisons of stenosis measurements between PETRA-MRA/TOF-MRA and reference standard DSA.

**Figure 3:**
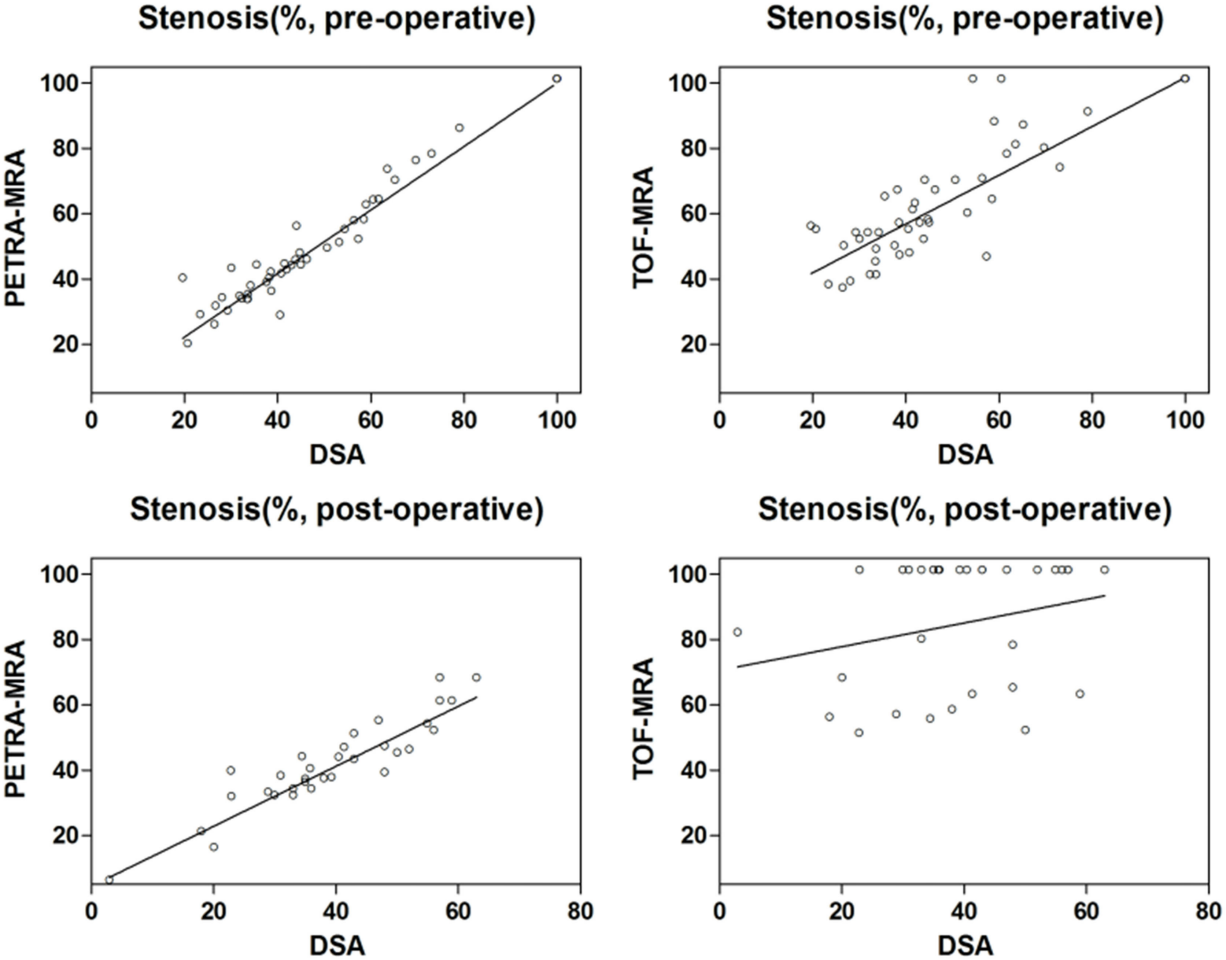
Spearman correlation plots for the comparisons of stenosis measurements between PETRA-MRA/TOF-MRA and reference standard DSA. For pre-intervention evaluation, there was a better correlation in measuring stenosis between PETRA-MRA (*r* = 0.96, *CV* = 4.6%) and DSA than between 3D-TOF-MRA (*r* = 0.85, *CV* = 11.0%) and DSA; For post-intervention evaluation, PETRA-MRA had an excellent agreement with DSA (*r* = 0.90, *CV* = 5.4%), while 3D-TOF-MRA had a poor agreement with DSA (*r* = 0.26, *CV* = 20.9%).

The agreement between MRAs and DSA for the degree of stenosis measurements is shown in Table 1, and the Bland-Altman plots and Spearman correlation plots are shown in Figures 2, 3. For pre-intervention evaluation, PETRA-MRA showed better agreement with DSA in measuring the degree of stenosis compared to 3D-TOF-MRA (*r* = 0.96 vs. *r* = 0.85 and measurement error/CV 4.6 vs. 11.0%). For post-intervention evaluation, PETRA-MRA had an excellent agreement with DSA (*r* = 0.90, *CV* = 5.4%), while 3D-TOF-MRA had a poor agreement with DSA (*r* = 0.26, *CV* = 20.9%). For both pre-and post-intervention, 3D-TOF-MRA overestimated the degree of stenosis compared to DSA (62.0 ± 25.6 vs. 54.5 ± 25.8%, *p* < 0.001; 84.9 ± 19.7 vs. 39.3 ± 13.6%, *p* < 0.001, respectively), while PETRA-MRA had comparable stenosis measurements with DSA (53.4 ± 27.3 vs. 54.5 ± 25.8%, *p* = 0.10; 40.6 ± 13.7 vs. 39.3 ± 13.6%, *p* = 0.18, respectively).

**Table 1:** Comparison of PETRA-MRA/TOF-MRA and DSA in measurements of stenosis.

	**Mean ± SD**	***CV* (%)**	***p*** ^*^	**Bias**	**LOA**	***r*** ^**#**^	***ICC***
**Stenosis (100%, Pre-operative)**							
DSA	54.5 ± 25.8	Reference	Reference	Reference	Reference	Reference	Reference
PETRA-MRA	53.4 ± 27.2	4.6	0.10	1.1	(−8.3, 10.5)	0.96	0.98
TOF-MRA	62.0 ± 25.6	11.0	<0.001	−7.5	(−29.0, 13.9)	0.85	0.87
**Stenosis (100%, Post-operative)**							
DSA	39.3 ± 13.6	Reference	Reference	Reference	Reference	Reference	Reference
PETRA-MRA	40.6 ± 13.7	5.4	0.18	−1.3	(−11.9, 9.3)	0.90	0.92
TOF-MRA	84.9 ± 19.7	20.9	<0.001	−45.6	(−86.7, −4.6)	0.26	0.05

* *Comparison between MRA and DSA*.

# *Spearman correlation*.

*SD, standard deviation; CV, coefficient of variation; LOA, limit of the agreement; ICC, intra-class coefficient*.

Using DSA as the reference standard, the sensitivity, specificity, PPV, and NPV of PETRA-MRA and 3D-TOF-MRA for detecting stenosis >50% and stenosis >75% are presented in Tables 2, 3. PETRA-MRA had overall better diagnostic performance than 3D-TOF-MRA when compared with DSA as the reference.

**Table 2:** Evaluation of the diagnostic performance of PETRA-MRA and TOF-MRA for detecting stenosis >50% and stenosis >75% using DSA as reference for the pre-intervention evaluation.

**Pre-operative**	**Detection of** **>50% stenosis**						**Detection of** **>75% stenosis**					
	**DSA** **(+)**	**DSA** **(-)**	**Sensitivity**	**Specificity**	**PPV**	**NPV**	**DSA** **(+)**	**DSA** **(–)**	**Sensitivity**	**Specificity**	**PPV**	**NPV**
PETRA-MRA (+)	23	0	95.8% (76.9–99.8)^*^	100.0% (85.4–100.0)	100.0% (82.2–100.0)	96.7% (80.9–99.8)	11	1	100.0% (67.9–100.0)	97.6% (85.9–99.9)	91.7% (59.8–99.6)	100.0% (89.3–100.0)
PETRA-MRA (–)	1	29					0	41				
TOF-MRA (+)	23	6	95.8% (76.9–99.8)	79.3% (59.7–91.3)	79.3% (59.7–91.3)	95.8% (76.9–99.8)	11	4	100.0% (67.9–100.0)	90.5% (76.5–96.9)	73.7% (44.8–91.1)	100.0% (88.6–100.0)
TOF-MRA (–)	1	23					0	38				

* *95% confidence interval shown*.

*PPV, positive predictive value; NPV, negative predictive value*.

**Table 3:** Evaluation of the diagnostic performance of PETRA-MRA and TOF-MRA for detecting stenosis >50% and stenosis >75% using DSA as a reference of the post-intervention.

**Post-operative**	**Detection of** **>50% stenosis**						**Detection of** **>75% stenosis**					
	**DSA** **(+)**	**DSA** **(–)**	**Sensitivity**	**Specificity**	**PPV**	**NPV**	**DSA** **(+)**	**DSA** **(–)**	**Sensitivity**	**Specificity**	**PPV**	**NPV**
PETRA-MRA (+)	7	3	87.5% (46.7–99.3)^*^	87.5% (66.5–96.7)	70% (35.4–91.9)	95.5% (75.1–99.8)	0	0	0	100% (86.7–100.0)	0	100% (86.7–100.0)
PETRA-MRA (–)	1	21					0	32				
TOF-MRA (+)	8	24	100.0% (59.8–100.0)	0 (0–17.2)	25% (12.1–43.8)	0	0	22	0	31.3% (16.7–50.1)	0 (0–18.5)	100.0% (65.5–100.0)
TOF-MRA (–)	0	0					0	10				

* *95% confidence interval shown. PPV, positive predictive value; NPV, negative predictive value*.

**Figure 4:**
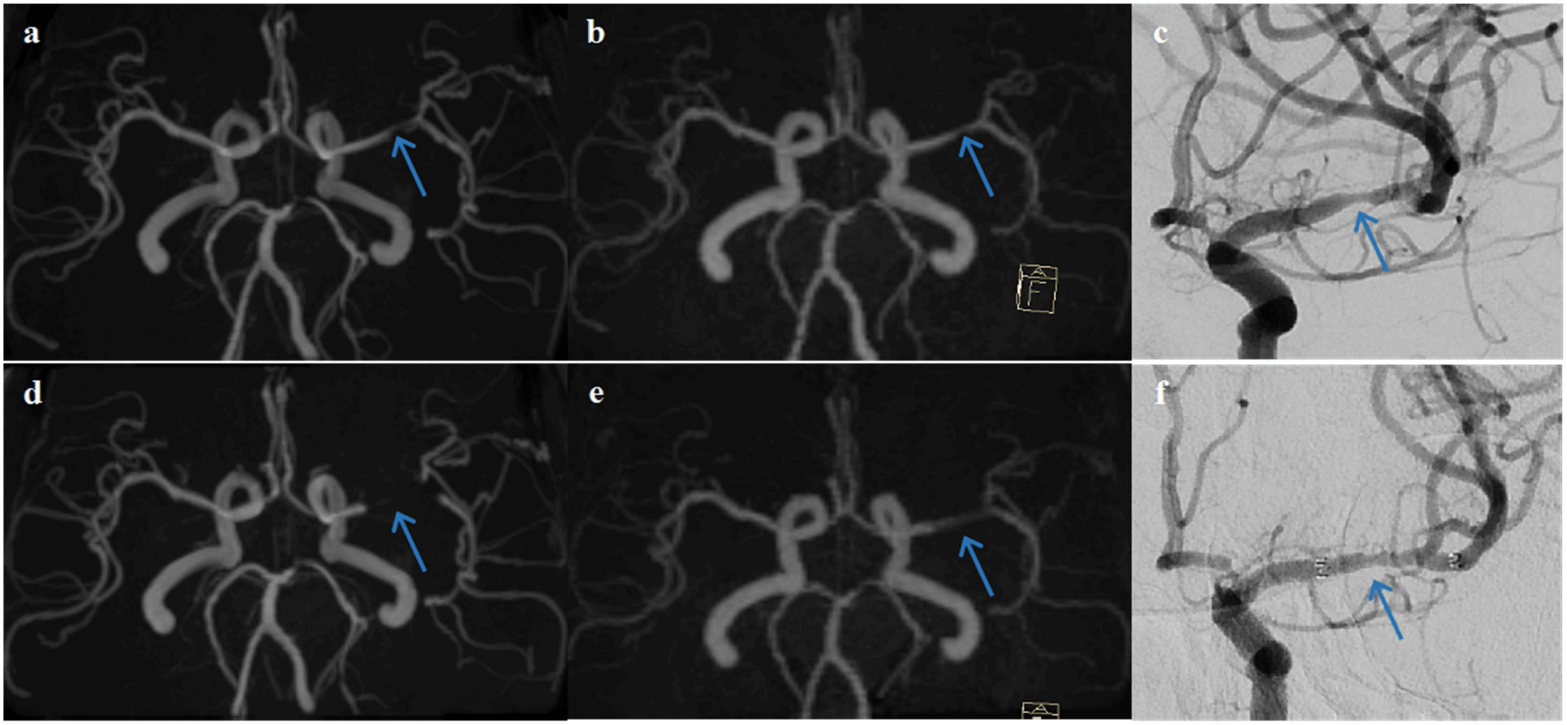
A representative case of a 63-year-old female patient who underwent stent angioplasty due to atherosclerotic stenosis of the left middle cerebral artery (MCA) at the M1 segment (blue arrows). Pre-intervention images are shown on the top row **(a)** TOF-MRA, **(b)** PETRA-MRA, and **(c)** DSA. The degree of stenosis was 79.8% on TOF, 65.3% on PETRA-MRA, and 68.6% on DSA. Image quality scores of the PETRA-MRA and 3D-TOF were both 4. Post-intervention images are shown on the bottom row **(d)** 3D-TOF, **(e)** PETRA-MRA, and **(f)** DSA. Post-intervention TOF-MRA had strong signal loss near the stent, and post-intervention PETRA-MRA had mild signal loss near the stent comparing with reference DSA. PETRA-MRA had significantly higher image quality scores than those of TOF-MRA considering flow visualization within the stents (PETRA-MRA, 4; 3D-TOF, 1) and susceptibility artifact (PETRA-MRA, 3; 3D-TOF, 1).

**Figure 5:**
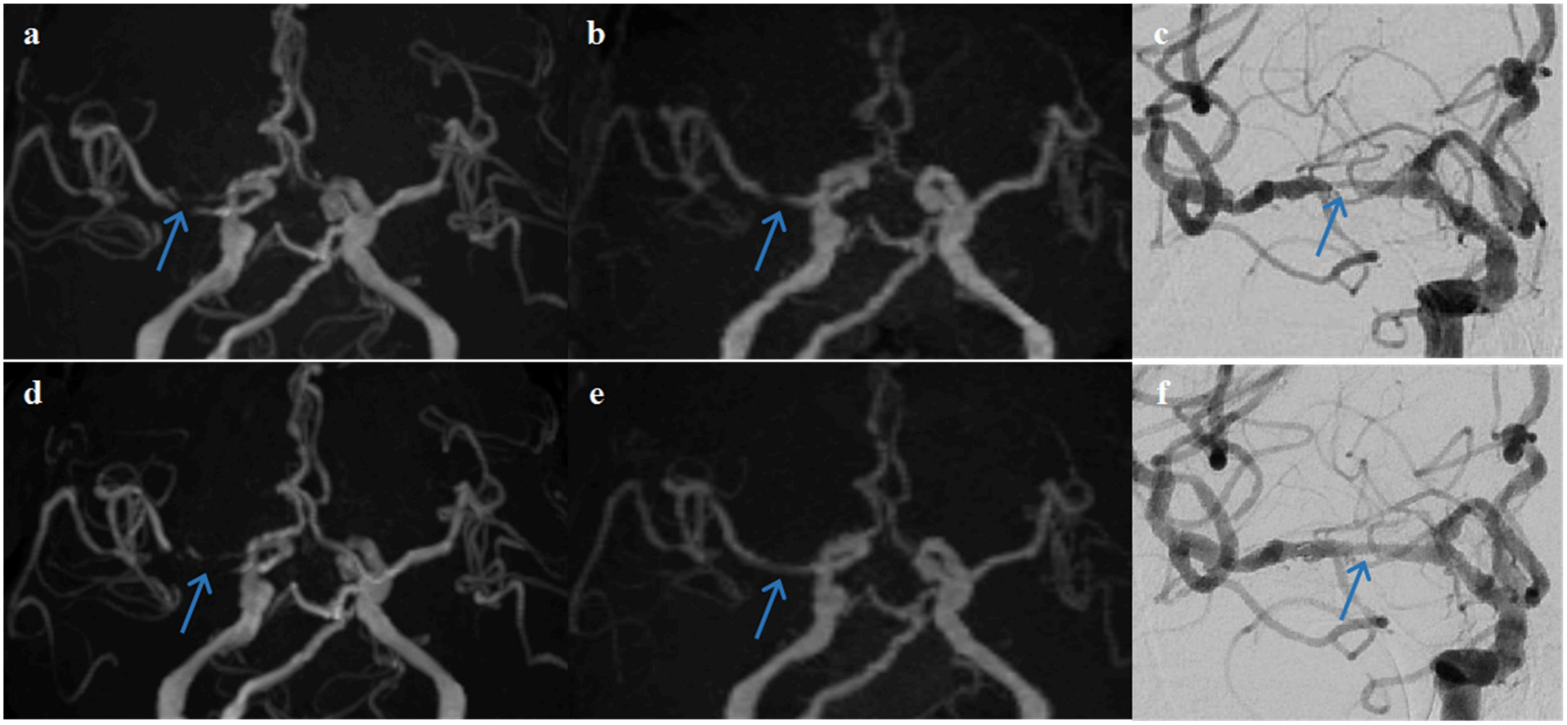
A representative case of a 68-year-old female patient who underwent stent angioplasty due to atherosclerotic stenosis of the right middle cerebral artery (MCA) at the M1 segment (blue arrows). Pre-intervention images are shown on the top row **(a)** TOF-MRA, **(b)** PETRA-MRA, and **(c)** DSA. The degree of stenosis was 100% on TOF (pseudo-occlusion), 79.8% on PETRA-MRA, and 86.3% on DSA. Image quality scores of the PETRA-MRA and 3D-TOF were 4 and 3, respectively. Post-intervention images are shown on the bottom row **(d)** 3D-TOF, **(e)** PETRA-MRA, and **(f)** DSA. Post-intervention TOF-MRA had strong signal loss near the stent, and post-intervention PETRA-MRA had minimal signal loss near the stent comparing with reference DSA. PETRA-MRA had significantly higher image quality scores than those of TOF-MRA considering flow visualization within the stents (PETRA-MRA, 4; 3D-TOF, 1) and susceptibility artifact (PETRA-MRA, 4; 3D-TOF, 1).

The examples from two patients having pre- and post-interventional imaging are shown in Figures 4, 5.

### Inter-Reader Agreement

For the pre-intervention image quality scoring, there was good agreement among the three readers for PETRA-MRA (ICC = 0.84), but a moderate agreement for 3D-TOF-MRA (ICC = 0.66). For the post-intervention image quality evaluation of flow visualization, there was a good inter-reader agreement for PETRA-MRA (ICC = 0.74) and 3D-TOF-MRA (ICC = 0.91). For the susceptibility artifacts scoring, there was a good inter-reader agreement for PETRA-MRA (ICC = 0.81) and 3D-TOF-MRA (ICC = 0.87) as well.

**Figure 6:**
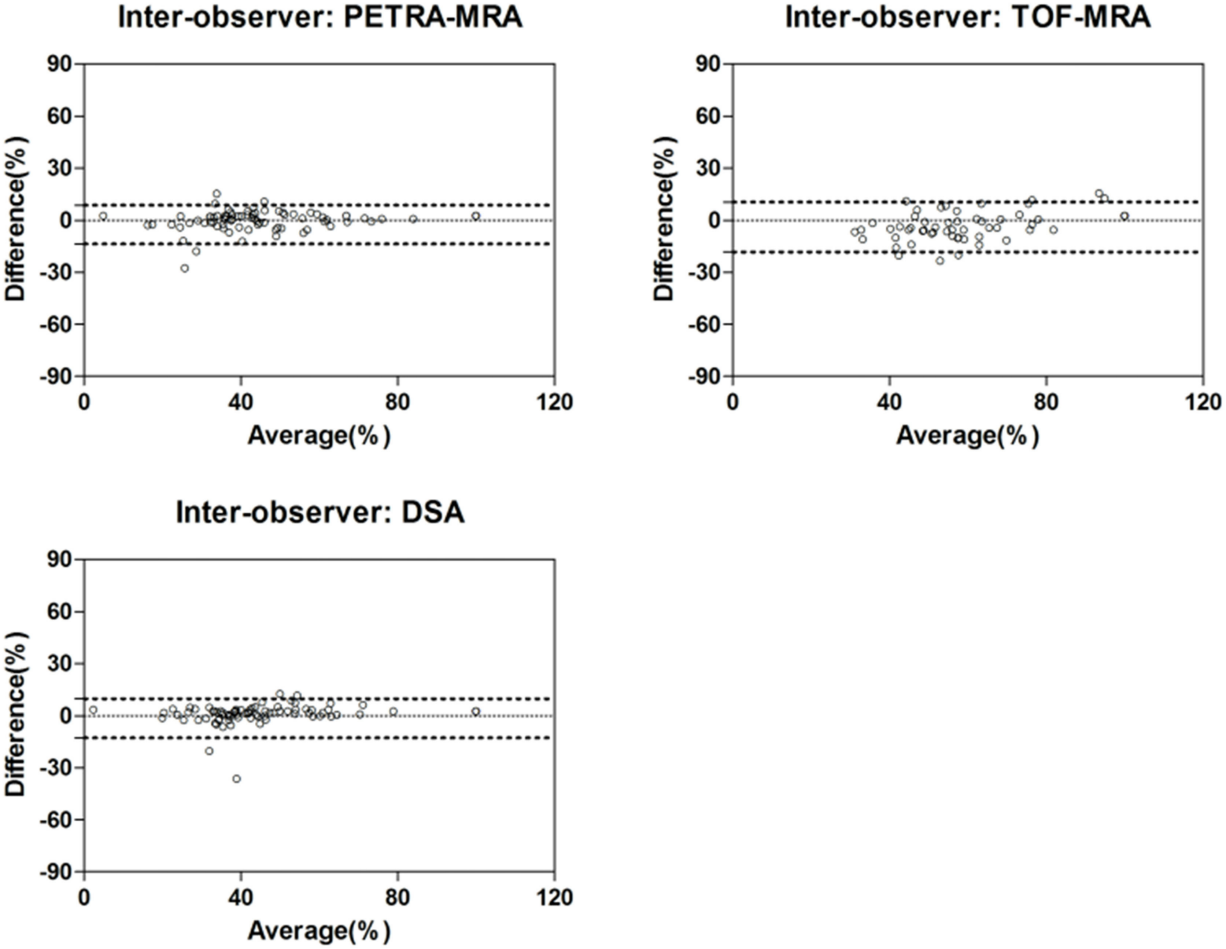
Inter-observer agreement of stenosis measurements using PETRA-MRA, TOF-MRA, and DSA.

There was an excellent inter-reader agreement for the measurement of the degree of stenosis with PETRA-MRA (ICC = 0.96), 3D-TOF-MRA (ICC = 0.94), and DSA (ICC = 0.96). The Bland-Altman plots are shown in Figure 6 and results are shown in Table 4.

**Table 4:** Summary of the inter-observer agreement for stenosis measurements.

**Stenosis (100%)**	**Reader 1**	**Reader 2**	***CV* (%)**	**Bias**	**LOA**	***ICC***
DSA	48.7 ± 23.1	50.2 ± 21.9	5.7	−1.5	(−12.8, 9.7)	0.96
PETRA-MRA	48.5 ± 23.8	51.1 ± 22.6	5.7	−2.5	(−13.7, 8.6)	0.96
TOF-MRA	70.6 ± 26.0	74.5 ± 22.0	7.4	−3.9	(−18.4, 10.5)	0.94

*CV, coefficient of variation; LOA, limit of the agreement; ICC, intra-class coefficient*.

## Discussion

This study demonstrated that PETRA-MRA had good image quality and accurate measurement of the MCA stenosis for both pre-and post-stenting comparing to gold standard DSA. The performance of PETRA-MRA was significantly better than 3D-TOF-MRA, especially after stent implementation. To our best knowledge, this was the first study using PETRA-MRA for the evaluation of intracranial stenosis post stent placement. Our study showed PETRA-MRA was a promising non-radiation non-contrast alternative to DSA for MCA stenosis quantification, and it was especially attractive for the routine surveillance of patients after stent employment when multiple scans were required.

The accurate quantification of the degree of MCA stenosis plays an important role in the management of stroke patients to decide the treatment strategy between medical therapy and intervention (3). Because of the invasive nature of the reference standard DSA, other non-invasive techniques including CTA, CE-MRA, and 3D-TOF-MRA are often used for the initial evaluation of the stenosis (5–7). 3D-TOF-MRA doesn't require the use of contrast and is free of radiation, which is promising for clinical use, but it is vulnerable to hemodynamic fluctuations which lead to signal voids (by phase dispersion) (7, 25). A recent study by Tian et al. (7) evaluated 176 intracranial artery segments with stenosis in the Circle of Willis and found 3D-TOF-MRA overestimated stenosis compared to 3D rotational DSA (65 ± 19 vs. 51 ± 15%, *p* < 0.001), and our results for the pre-intervention evaluation reported similar findings. Compared with 3D-TOF-MRA with a TE value around 3–5 ms, PETRA-MRA had an ultra-short TE of <100 μs. Therefore, PETRA-MRA was less sensitive to turbulent flow artifacts around the stenotic site.

A recent study by Shang et al. included 26 patients with intracranial stenosis and compared the stenosis evaluation of PETRA-MRA with 3D-TOF-MRA using CTA as a reference (21). They found PETRA-MRA had better image quality than 3D-TOF-MRA for signal homogeneity and venous signal suppression, and PETRA-MRA had a better agreement for stenosis categorization (<30, 30–69, 70–99, 100%) with CTA than 3D-TOF-MRA had with CTA (kappa, 0.90 vs. 0.81). Our results agreed with their findings with several advantages: (1) we had a large sample size than theirs (*n* = 53 vs. *n* = 26); (2) we used DSA as the reference standard while they used CTA as a reference; (3) we performed quantitative stenosis measurements while they only did qualitative grading.

Future comparison of PETRA-MRA to other promising advanced imaging techniques for stenosis measurement and post-intervention evaluation may prove valuable to determine the ideal imaging approach. Vessel wall MRI has proven to be a valuable technique for intracranial stenosis evaluation (7, 26, 27). Tian et al. found 3D vessel wall MRI agreed better with DSA than 3D-TOF-MRA (*r* = 0.91 vs. *r* = 0.70). Compared with bright blood MRA, black blood MRI did not provide easy visualization of stenosis, but it could characterize vessel wall features related to plaque vulnerability (28–30).

Intracranial angioplasty and stenting are beneficial for selected patient cohorts and have been used widely in many experienced centers (9, 31). After stent placement, imaging surveillance is necessary to monitor for in-stent restenosis (12). Computed tomography angiography is limited for post-stenting evaluation due to metal beam hardening artifact obscuring luminal evaluation (14). Traditional MRA methods including CE-MRA and 3D-TOF-MRA are limited due to susceptibility artifacts that create signal voids around the stent, overestimating the degree of stenosis (15, 32). To the best of our knowledge, no studies have been published evaluating PETRA-MRA for post-stenting evaluation of intracranial stenosis. However, there have been a few studies evaluating PETRA-MRA for stent-assistant coiling for intracranial aneurysms (15, 20). You et al. evaluated 61 patients who underwent stent-assisted coiling of an intracranial aneurysm with zero-TE MRA (a technique comparable to PETRA-MRA), CE-MRA, 3D-TOF-MRA, and DSA (15). They found zero-TE MRA had the best visualization of in-stent flow and performed better than CE-MRA and 3D-TOF-MRA. Our study had similar findings for post-stenting of intracranial stenosis. The ultra-short TE or zero TE sequences, in principle, begin the signal acquisition nearly immediately after the excitation when the phase dispersion within the voxel is still minimal, thus there is little image distortion and signal loss (33). As PETRA-MRA is a non-invasive and non-radiation technique, it has great potential for the follow up of patients after intracranial stent placement.

Our study has several limitations. First, this is a single-center study with a small sample size for post-stenting evaluation. Future larger-scale multi-center studies are required to confirm our findings. Second, we only evaluated MCA stenosis, and the performance of PETRA-MRA in other intracranial arteries is warranted. Third, the PETRA-MRA sequence has a long scan time relative to 3D-TOF-MRA. Future evaluation of the impact the PETRA-MRA has on MRI workflows relative to the use of 3D-TOF-MRA would be helpful to better determine the feasibility of its utilization in standard imaging algorithms.

## Conclusions

PETRA-MRA is accurate and reproducible for quantifying MCA stenosis both pre- and post-stenting compared with DSA and performs better than 3D-TOF-MRA.

## Data Availability Statement

The original contributions presented in the study are included in the article/supplementary material, further inquiries can be directed to the corresponding author/s.

## Ethics Statement

The studies involving human participants were reviewed and approved by The First Affiliated Hospital of Zhengzhou University. The patients/participants provided their written informed consent to participate in this study. Written informed consent was obtained from the individuals for the publication of any potentially identifiable images or data included in this article.

## Author Contributions

FZ, YR, MZ, XG, JC, and CZ conceived and designed the experiments. FZ, YR, MZ, XL, JN, and XW performed the experiments. FZ, YR, and CZ analyzed the data. FZ, YZ, SL, JZ, and MM-B participated in the completion of the manuscript. All authors have made significant scientific contributions to this manuscript and reviewed the manuscript.

## Funding

The study was supported by the medical and technology grant of Henan province (Grant Number 2018020137). CZ was supported by National Institute of Health (NIH) grant (Grant Number R00HL136883).

## Conflict of Interest

JZ is employed by Siemens. The remaining authors declare that the research was conducted in the absence of any commercial or financial relationships that could be construed as a potential conflict of interest.

## Publisher's Note

All claims expressed in this article are solely those of the authors and do not necessarily represent those of their affiliated organizations, or those of the publisher, the editors and the reviewers. Any product that may be evaluated in this article, or claim that may be made by its manufacturer, is not guaranteed or endorsed by the publisher.
